# Musicians do not benefit from differences in fundamental frequency when listening to speech in competing speech backgrounds

**DOI:** 10.1038/s41598-017-12937-9

**Published:** 2017-10-03

**Authors:** Sara M. K. Madsen, Kelly L. Whiteford, Andrew J. Oxenham

**Affiliations:** 1 Hearing Systems, Department of Electrical Engineering, Technical University of Denmark, Ørsteds Plads 352, 2800 Kgs. Lyngby, Denmark; 2 Department of Psychology, University of Minnesota, 75 East River Parkway, Minneapolis, MN 55455 USA

## Abstract

Recent studies disagree on whether musicians have an advantage over non-musicians in understanding speech in noise. However, it has been suggested that musicians may be able to use differences in fundamental frequency (F0) to better understand target speech in the presence of interfering talkers. Here we studied a relatively large (N = 60) cohort of young adults, equally divided between non-musicians and highly trained musicians, to test whether the musicians were better able to understand speech either in noise or in a two-talker competing speech masker. The target speech and competing speech were presented with either their natural F0 contours or on a monotone F0, and the F0 difference between the target and masker was systematically varied. As expected, speech intelligibility improved with increasing F0 difference between the target and the two-talker masker for both natural and monotone speech. However, no significant intelligibility advantage was observed for musicians over non-musicians in any condition. Although F0 discrimination was significantly better for musicians than for non-musicians, it was not correlated with speech scores. Overall, the results do not support the hypothesis that musical training leads to improved speech intelligibility in complex speech or noise backgrounds.

## Introduction

Musical training can enhance some abilities related to music, such as pitch^1–4^, rhythm^5,6^, and melody^7^ discrimination. It remains unclear if musical training transfers to less directly related abilities, such as speech perception in noise. This question is important because understanding speech in noise is a crucial aspect of our ability to communicate in social settings and in other noisy environments. The challenge of communicating in noise increases with age and with hearing loss^8,9^, making it all the more important to find ways to improve this skill.

Several studies have compared the speech understanding abilities of highly trained musicians and non-musicians for sentences in noise^10^. Some studies have found no significant difference between groups^4,11,12^, while the differences reported as significant in other studies have generally been small^13–15^. Similar inconsistencies have been found for studies investigating differences between musicians and non-musicians in their ability to understand speech in a background of competing speech. Using the QuickSIN test^16^, a clinical test where syntactically correct sentences without context (target) are presented in a background of four-talker babble, some studies reported an advantage for musicians^5,14,17,18^, whereas another did not^4^. Other studies used speech from a single talker to mask the target sentences. Of these, one study found a difference^19^ and two did not^11,20^. Finally, two studies using the same test, and some of the same participants, with speech sentence materials taken from a closed (matrix) set of words for both the target and two maskers^21,22^, found no group differences when the maskers and targets were co-located in front of the listener, but a significant advantage for the musician group when the target was presented from the front and the maskers were presented at ±15° for conditions with natural speech maskers. In reviewing this mixed literature, no clear pattern emerges to explain why some studies find small but significant effects, whereas others do not.

At least some of the musician advantage observed in some studies may be explained by enhanced cognitive abilities^17,21^. For example, several studies have found better auditory working memory in musicians than in non-musicians^17,20,21,23,24^ and two studies have found a correlation between working memory and speech in noise scores^17,21^. However, other studies found auditory working memory to be similar for groups of musicians and non-musicians^11,25^. It is also possible that the musician advantage is related to the enhanced pitch discrimination abilities previously found in musicians^1–3^. In one study, the target was distinguished from the masker talker by introducing differences in either fundamental frequency (F0, the acoustic correlate of pitch) or vocal tract length, and an advantage was observed in all conditions^19^. The fact that an advantage was observed even in a condition with no average F0 or vocal tract length difference might be explained by the enhanced ability of musicians to take advantage of momentary differences in F0 between the target and masker, even though the average F0s were the same.

The aim of the present study was to determine whether musicians are more able than non-musicians to use differences in F0 between competing speakers. Given the mixed results of previous studies, we recruited a larger number of subjects (60) than has typically been tested in such experiments. To explore the role of momentary F0 differences, we used speech that either had natural F0 variations or had been monotonised to remain on the same F0 throughout the sentence. If musicians rely on momentary differences in F0, then their advantage should be reduced or eliminated in the case of monotone sentences presented with the same F0. Similarly, if musicians are better able to make use of small differences in F0 between competing speakers, then their advantage should be greatest at small F0 differences between the speakers, and should be related to their ability to discriminate small differences in F0. These predictions were tested by measuring musicians’ and non-musicians’ speech intelligibility in the presence of two-talker maskers and speech-shaped noise. The speech was manipulated to introduce systematic differences in the average F0 between the target and masker talkers with either natural F0 variations or a monotone F0. The study also included an F0 discrimination task and measures of cognitive ability. The results from our study suggest no clear benefit of musical training on speech perception in any of the conditions tested, and no relationship with F0 discrimination abilities.

## Results

### F0 discrimination

**Figure 1:**
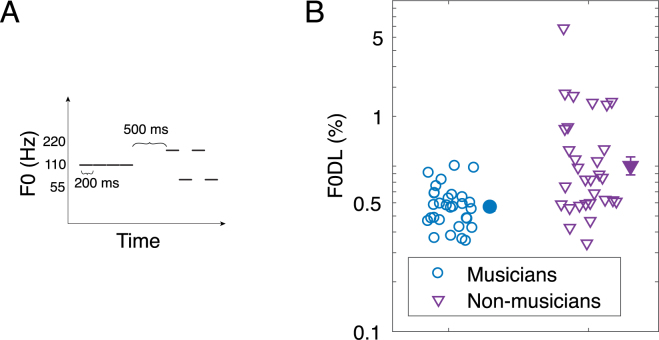
Fundamental frequency difference limens (F0DLs) for musicians and non-musicians. (**A**) Schematic diagram of the stimuli used in the experiment. (**B**) Individual thresholds for each participant (open symbols) are shown, along with group means (solid symbols). Errors bars represent ±1 standard error of the mean across participants.

F0 discrimination was tested for a harmonic complex tone with an F0 of 110 Hz, corresponding to the average F0 of the target speech. In line with earlier studies^1–4^, discrimination thresholds were significantly lower (better) for the musicians than the non-musicians (t-test on independent samples with unequal variance; *t*_58_ = 4.27, *p* < 0.001, Cohen’s *d* = 1.10), although there was considerable between-subject variability and overlap between the two groups (Fig. 1B). The group difference was still significant when excluding the non-musician with the highest (poorest) threshold (*t*_57_ = 4.26, *p* < 0.001, Cohen’s *d* = 1.11).

### Speech intelligibility

Speech intelligibility was tested using sentences from the Hearing in Noise Test (HINT)^26^ with a male talker that had either natural F0 fluctuations or was processed to remain on a single F0 throughout each sentence. The masker was either a Gaussian noise, spectrally shaped to have the same long-term spectrum as the target speech, or a two-talker masker, also with the same long-term spectrum as the target speech. The type of F0 contour of the two-talker masker was always the same as that of the target speech, being either natural or monotone. Speech intelligibility was measured as proportion of words reported correctly in each condition, considering all words except “the” and “a/an”. Obvious misspellings or homophones were considered correct.

**Figure 2:**
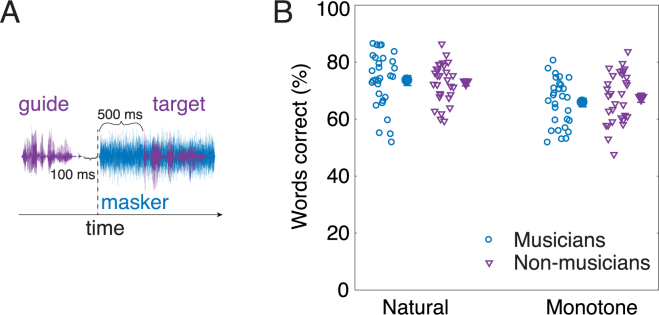
(**A**) Schematic diagram of the stimuli used in the experiment. (**B**) Proportion of words correctly identified for natural and monotone speech masked by speech-shaped noise. Individual speech scores (open symbols) and means across participants in each group (solid symbols). Error bars indicate ±1 standard errors of the mean.

The results for natural and monotone speech in the presence of speech-shaped noise showed that speech intelligibility was very similar for the two listener groups and for natural and monotone speech (Fig. 2B). This similarity between groups was confirmed by a mixed-model analysis of variance (ANOVA) with proportion of words correct transformed into rationalized arcsine units (RAU)^27^ as the dependent variable, listener group (musicians and non-musicians) as the between-subjects factor, and speech contour (natural and monotone) as the within-subjects factor. The difference between the natural and monotone speech was significant (F_1,58_ = 25.2, p < 0.001, ${\eta }_{G}^{2}$ = 0.14), in line with earlier studies of the effects of natural F0 variations on speech intelligibility^28,29^. However, neither the main effect of listener group (F_1,58_ = 0.060, p = 0.81, ${\eta }_{G}^{2}$ < 0.001) nor the interaction between listener group and speech contour (F_1,58_ = 0.98, p = 0.33, ${\eta }_{G}^{2}$ = 0.006) was significant. The marginally significant difference between the two groups in terms of IQ (see Table 1) suggested some possible relationship between IQ and measures of auditory or speech performance. However, there was no significant correlation between speech scores in noise and our combined measure of IQ (R^2^ = 0.0067, p = 0.27). Appropriate one-tailed tests were used here and below.

**Table 1:** Overview of demographic averages for 30 musicians and 30 non-musicians. Standard deviations﻿ are in parentheses. Table also show p-values for comparison of the two groups. Independent-samples t-tests were used to compare age and IQ, and a χ^2^ test was used to compare distribution of gender distribution in the two groups.

	Musicians (n = 30)	Non-musicians (n = 30)	p-value
Age (years)	21.13 (2.47)	20.9 (2.70)	0.73
Sex	21 females, 9 males	22 females, 8 males	0.77
IQ	118.9 (10.9)	113.5 (10.32)	0.054

**Figure 3:**
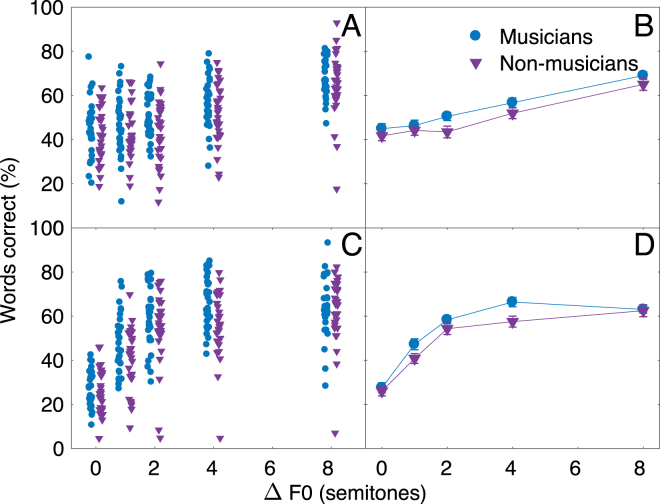
Proportion of words correctly identified for natural speech (**A** and **B**) and monotone speech (**C** and **D**) as a function of difference in overall mean fundamental frequency (ΔF0) of target and maskers. (**A** and **C**) Speech scores for each participant. (**B** and **D**) Means across participants in each group. Error bars indicate ±1 standard error of the mean.

For conditions where the target speech was presented in a background of a two-talker masker, scores increased with increasing ΔF0 between the target and masker in conditions with natural F0 contours (a and b), and with a monotone F0 (c and d). However, the pattern of improvement with increasing ΔF0 seemed different in the two cases, with a more rapid and then saturating improvement in the case of the monotone speech and a more gradual improvement with the natural F0 variations. This outcome is expected, given that the instantaneous F0s of the natural speech overlap over a wide range of ΔF0s, meaning that a large mean difference in F0 is required to achieve complete F0 separation between the target and masker. There also appeared to be a small effect of musical training, with average musicians’ scores lying slightly above those of the non-musicians in most conditions. This is also the case when excluding the non-musician with the worst performance. These impressions were tested in a mixed-model ANOVA on the RAU-transformed scores, with within-subject factors of ΔF0 (0–8 semitones) and F0 contour (natural or monotone), and a between-subjects factor of musical training (musician or non-musician). Speech-on-speech scores, averaged across all conditions, were not correlated with our combined measure of cognitive ability (N_60_: R^2^ = 0.039, p = 0.065; N_59_: R^2^ = 0.011, p = 0.21) when excluding one non-musician who otherwise drove the correlation. Therefore, this measure was not included as a covariate. The ANOVA confirmed a significant effect of ΔF0 (F_4,232_ = 171.2, p < 0.001, ${\eta }_{G}^{2}$ = 0.39), and a significant interaction between F0 and speech contour (F_4,232_ = 45.06, p < 0.001, ${\eta }_{G}^{2}$ = 0.12). However, the main effect of musical training did not reach significance (F_1,58_ = 3.27, p = 0.076, ${\eta }_{G}^{2}$ = 0.03). In addition, neither the two-way interactions between musical training and ΔF0 (F_4,232_ = 1.29, p = 0.28, ${\eta }_{G}^{2}$ = 0.005) or between musical training and speech contour (F_1,58_ = 0.064, p = 0.80, ${\eta }_{G}^{2}$ = 0.0001), nor the three-way interaction between musical training, speech contour, and ΔF0 approached significance (F_4,232_ = 1.48, p = 0.21, ${\eta }_{G}^{2}$ = 0.004). This outcome shows that the speech intelligibility of musicians and non-musicians did not differ significantly in any of the conditions tested. The lack of significance is consistent with the general overlap in the performance of the two groups, shown in Fig. 3A and C.

**Figure 4:**
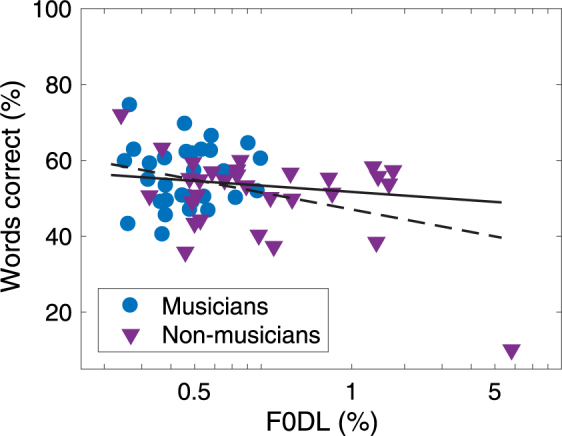
Proportion of correct words as a function of F0 difference limens. Speech scores were averaged across all conditions with overall or momentary F0 differences between target and maskers. Regression lines are shown for all participants (dashed line) and for 59 participants (solid line).

So far, this analysis has provided no evidence that musical training facilitates the ability to use cues from differences in F0 for understanding speech in the presence of competing speech. It is, however, possible that this ability is related to pitch discrimination abilities, which in this and many other studies have been shown to be enhanced by musical training^1–3^. The RAU-transformed individual speech scores were averaged across all conditions with overall or instantaneous F0 differences between target and maskers, i.e. all conditions with speech maskers except the monotone condition with ΔF0 = 0. The correlation (Fig. 4) was not significant (R^2^ = 0.024, p = 0.12) when excluding one non-musician who otherwise drove the correlation (R^2^ = 0.15, p = 0.001).

The group of musicians was also considered alone to explore the effects of age of onset of musical training and years of training on speech intelligibility. The addition of age of onset as a covariate showed that there was no effect of age of onset (F_1,28_ = 0.013, p = 0.91, ${\eta }_{G}^{2}$ < 0.001), and no interaction between age of onset and speech contour (F_1,28_ = 0.32, p = 0.57, ${\eta }_{G}^{2}$ = 0.001) or ΔF0 (F_4,112_ = 1.08, p = 0.37, ${\eta }_{G}^{2}$ = 0.009), or between age of onset, speech contour, and ΔF0 (F_4,112_ = 1.81, p = 0.13, ${\eta }_{G}^{2}$ = 0.01). Similarly, when adding years of training as a covariate instead, there was no main effect of years of training (F_1,28_ = 0.15, p = 0.70, ${\eta }_{G}^{2}$ = 0.002) and no interaction between years of training and speech contour (F(1, 28) = 0.078, p = 0.78, ${\eta }_{G}^{2}$ < 0.001), between years of training and ΔF0 (F_4,112_ = 0.81, p = 0.52, ${\eta }_{G}^{2}$ = 0.007), or between years of training, speech contour, and ΔF0 (F_4,112_ = 0.65, p = 0.63, ${\eta }_{G}^{2}$ = 0.004). Hence, neither number of years of musical training nor the age of onset of training was related to the ability to make use of F0 differences for understanding masked speech.

In addition to F0 discrimination, members of the musician group were also asked to identify musical intervals. Performance in this musical interval identification task was not correlated with individual averaged speech scores for either the noise masker (Spearman’s S = 5027.7, p = 0.27) or the speech masker (Spearman’s S = 4241.5, p = 0.62). The fact that nine musicians did not major in music at university level might also mean that the musicians in this study were less experienced than musicians in some other studies. However, even when only including the 21 music majors in the musician group, neither the main effect of musical training (F_1,49_ = 3.63, p = 0.063, ${\eta }_{G}^{2}$ = 0.04) nor the interactions between musical training and ΔF0 (F_4,196_ = 1.33, p = 0.26, ${\eta }_{G}^{2}$ = 0.006), between musical training and speech contour (F_1,49_ = 0.23, p = 0.63, ${\eta }_{G}^{2}$ < 0.001) or between musical training, speech contour, and ΔF0 (F_4, 196_ = 1.48, p = 0.21, ${\eta }_{G}^{2}$ = 0.005) were significant. Nevertheless, our combined measure of cognitive ability was significantly correlated with years of musical training (R^2^ = 0.12, p = 0.033).

In summary, no significant enhancement of speech perception was found to relate to musical training in any of the conditions tested. A small benefit of musical training was seen in some conditions with differences between average or momentary target and masker F0. However, none of these differences reached statistical significance (p = 0.05), despite the relatively large number of subjects tested (N = 60). In addition, speech scores were not strongly related to our measure of cognitive ability or to F0 discrimination thresholds, or (when considering only musicians) to number of years of training or age of onset of musical training.

## Discussion

This study found no evidence that musicians are better than non-musicians at using differences in momentary or average F0 between competing speakers for understanding speech. The lack of effect of musical training on the improvement in performance with increasing F0 difference between the target and maskers is consistent with the findings of Baskent and Gaudrain^19^. However, in contrast to those earlier findings, no overall benefit of musical training was observed in our data. Nevertheless, mean overall performance was slightly but not significantly better for the musicians in the case of the speech masker. To better understand the extent to which the conclusions are limited by statistical power, the sample size needed to obtain a significant effect for the conditions with speech maskers was calculated from the estimated effect size, $f=\sqrt{{\eta }^{2}/(1-{\eta }^{2})}$ ^30^, where the generalized eta squared ${\eta }_{G}^{2}$ was used to estimate ${\eta }^{2}$ as recommended by Bateman^31^. Assuming the observed effect size is an accurate estimate for the population, and that the correlation among repeated measures is 0.5, a sample size of 148 (n = 74 in each group) would be required to obtain a significant difference between groups with statistical power at the recommended 0.80 level. This and the sample size of 230 participants estimated by Boebinger *et al*.^11^ suggest that a much larger number of participants is needed to detect these group differences than has been used in any previous study. Therefore, it is unlikely that the significant group difference found in some studies can be explained solely by differences in statistical power across studies. Indeed, to our knowledge, our sample of 60 is the largest tested to date on the question of whether musical training enhances speech perception in young adults. The estimated need for well over 100 participants to detect an effect of the size found here and in previous studies emphasizes the main point that effects of musicianship on speech perception, if present, tend to be so small as to render their importance in everyday life questionable. However, it has been suggested that musical training may counteract the negative effects of ageing on speech perception in noise^18^. Because our participants were all young, with a mean age of 21 years, our study cannot address this interesting possibility.

Although we attempted to match the two groups in terms of age, sex, and IQ, the difference in IQ between the two groups just failed to reach statistical significance (p = 0.054), with the musicians’ mean IQ being higher by 5.4 points. This difference is unlikely to have affected outcomes because, as reported in the Results, IQ was not correlated with speech perception in either noise or the two-talker masker. In addition, if anything, the higher mean IQ of the musicians may have led to an overestimate of the effects of musical training on speech perception.

Differences in average musical experience of participants between studies might explain some of the differences in conclusions. This study used similar criteria to define the musicians and slightly stricter criteria for non-musicians than other similar studies. Some studies^11,15,17,32^ only used musicians whose training started before the age of 7, whereas this study also included musicians who started at the age of 7. However, the lack of correlation between age of onset of training and speech scores in our data make this difference an unlikely explanation for the outcomes. Another potentially important variable that has rarely been examined is the degree of musical skill exhibited by the musicians. Our proxy measure was musical interval identification, which has been used in at least one earlier study^33^. Here again, no relationships were observed between musical skill and speech perception. Finally, if it were the case that only highly skilled or professional musicians exhibited an effect, this would severely limit the utility of musical education or training as a widespread intervention to improve speech perception in noise or other backgrounds.

As mentioned in the introduction, two studies from the same group^21,22^ found relatively large effects of musicianship in cases with natural speech, where the target and speech masker were spatially separated. However, one of these studies^22^ also showed that the effect of musicianship on spatially separated targets and maskers was abolished for time-reversed (unintelligible) maskers. It remains unclear what distinguishes these studies from ours and others that found small or no effects. One possibility relates to their use of a matrix sentence test, which uses a closed set of a limited number of words. It may be that musicians are better able to distinguish fine spectro-temporal details from a limited set of speech stimuli in tasks that use target and masker sentences with the same sentence structure. If so, it is not clear how well such results will generalize to more natural speech settings. In any case, a direct comparison of different speech tests in the same populations would be required to test this hypothesis.

In summary, we observed no significant effect on musical training on any of our measures, with the exception of F0 discrimination, despite a relatively large sample size of 60. Taken together with the generally small effects observed even in those studies that have reported a significant effect of musicianship, this outcome suggests that the effects of musical training are sufficiently fragile as to render questionable the relevance of musical training as a tool to enhance speech perception, at least among younger listeners.

## Methods

### Participants

**Figure 5:**
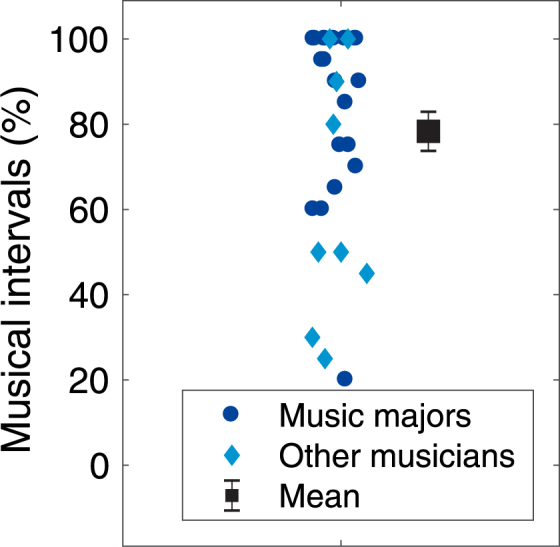
Proportion correct responses in the musical interval identification test.

30 musicians and 30 non-musicians participated in the study. The two groups were matched in age, sex, and IQ (Table 1). Cognitive ability (IQ) was measured using the Vocabulary and Matrix Reasoning subtests of the Wechsler Abbreviated Scale of Intelligence – Second Edition (WASI-II)^34^. The full-scale score was calculated by combining the scores from the two subtests. Participants in the musician group started training at or before the age of seven, had at least 10 years of musical training and currently played or sang for at least 5 hours a week. Non-musicians were required to have played an instrument or sung in an organized setting such as a choir for less than two years and to not have done so within the last 7 years. These criteria are comparable to those used in similar studies^17,19^. Details of the musical experience of the musicians can be found in Table 2. As a measure of degree of musical expertise, musical interval identification was measured for the musicians in a computerized test where they were asked to identify 20 musical intervals played sequentially on a piano. The participants could choose to repeat each interval once. Performance was variable even for the university music majors (Fig. 5) and was not related to number of years of musical training (R^2^ = 0.0034, p = 0.76).

**Table 2:** Overview over the musical experience of the musicians participating in the experiment.

	Age of onset (yr.)	Years of training	Primary instrument
1	6	12	piano
2	6	10	piano
3	6	10	voice
4	5	16	piano
5	5	17	piano
6	4	13	horn
7	6	10	voice
8	6	17	clarinet
9	5	15	oboe
10	5	18	cello
11	5	15	voice
12	4	16	voice
13	5	20	piano
14	4	17	violin
15	6	17	voice
16	4	15	violin
17	6	12	violin
18	6	13	piano
19	7	11	voice
20	5	13	piano
21	6	12	percussion
22	7	19	flute
23	6	14	oboe
24	7	13	voice
25	6	17	piano
26	5	14	piano
27	4	16	piano
28	4	17	piano
29	6	12	guitar
30	6	16	violin

All participants were native speakers of American-English and had audiometric thresholds of no more than 20 dB HL at octave frequencies between 250 and 8000 Hz. All subjects provided informed consent prior to their participation in the experiments. The experimental protocols were approved by the Institutional Review Board of the University of Minnesota and carried out in accordance with the corresponding guidelines and relevant regulations on the use of human subjects.

### F0 discrimination

F0 difference limens were measured using a 2-interval 2-alternative forced-choice paradigm similar to the one previously used for temporal fine structure assessment^35^ in order to simplify the task and reduce the need for training. A three-down, one-up adaptive procedure was employed that tracks the 79.4% correct point on the psychometric function^36^. Each interval contained a sequence of four 200-ms tones, gated on and off with 20-ms raised-cosine ramps, and presented immediately after each other. All tones in the reference interval had a F0 of 110 Hz which approximates the mean F0 of the target talker in the speech task. In the other interval, the F0 of the first and the third tone was higher and the F0 of the second and fourth tone was lower than that of the reference tones. The difference in F0 between the high and low tones was varied adaptively and geometrically centred on 110 Hz. A schematic diagram of the stimuli is provided in Fig. 1A. The participants were asked to identify the interval that contained the changes in pitch. Feedback was provided after each trial. Each tone contained all harmonics up to 6000 Hz and was filtered to have same spectral envelope as the long-term spectrum of the target HINT sentences^26^. The tones were presented at a level of 60 dB SPL. The two intervals were separated by a 500-ms interstimulus interval.

### Speech perception

Speech intelligibility was measured for natural and monotone speech masked by noise or by two speech maskers. HINT sentences^26^ spoken by a male speaker were used as the target and IEEE sentences^37^ spoken by another male speaker were used as speech maskers. In the two noise conditions, either natural or monotone speech was masked by noise. In half of the speech-on-speech conditions, a target of natural speech was masked by natural speech and the F0 of the masker sentences was manipulated in PRAAT^38^ so that the overall average F0 of the IEEE sentences was 0, 1, 2, 4, or 8 semitones lower than that of the HINT sentences. In the other half of the speech-on-speech conditions, both the target and the maskers were monotonised. The target sentence F0 was set to equal to the long-term average F0 of the HINT sentences and the F0 of the masker sentences was 0, 1, 2, 4, or 8 semitones lower than the target F0. Both noise and speech maskers were spectrally shaped to have the same long-term spectrum as the target sentences. The speech maskers were created by concatenating all IEEE sentences, then removing all gaps exceeding 100 ms, and finally dividing the concatenated sentences into blocks of 3.06 s. The maskers were gated with 50 ms raised-cosine onset and offset ramps and started 500 ms before and ended at least 100 ms after the target. The target sentences had a duration between 1.17 and 2.46 s. The participants were trained with 18 sentences, half of which were monotone and half were natural. For the training, the mean F0 of the target and masker were separated by 6 semitones. Immediately before each training and test trial, the participants would hear a HINT sentence (always the same sentence) in quiet to guide them toward the target voice (Fig. 2A). The speech in the guide sentence was natural for the natural speech conditions and monotone for the monotone speech conditions. Both target and each masker were presented at a level of 60 dB SPL resulting in an overall signal-to-masker ratio of −3 dB. The participants were told to listen for the voice of the guide sentence and were asked to type what they heard, exactly as they heard it, after each trial. Each of the 12 conditions was tested with 10 sentences in each of two blocks, for a total of 240 sentences. The order of the conditions was randomized within each block and was different for all participants within each group but was the same for the two groups. In other words, the order used for the first musician was the same as that used for the first non-musician. The participants were asked to take a small break between the two blocks. The total duration of the experiments for each subject was 3 hours on average. The sounds were generated with a Lynx Studio Technology L22 sound card at 24-bit resolution and were presented binaurally (diotically) via Sennheiser HD650 headphones to listeners seated within a sound-attenuating booth.

### Statistical analysis

The proportion of words correct was transformed into rationalized arcsine units (RAU)^27^ and analysed with a mixed-model ANOVA. Assumptions of normality of residuals were met for all ANOVAs described. Requirements of sphericity were fulfilled, as determined by Mauchly’s test of sphericity. The generalized eta-squared, ${\eta }_{G}^{2}$, was reported for all ANOVAs as a measure of effect size^31^. Non-parametric tests were used when the data failed to meet normality criteria.

### Data Availability

The datasets generated and analysed during the current study are available from the corresponding author upon reasonable request.
